# *Bacillus amyloliquefaciens* strain MBI600 induces salicylic acid dependent resistance in tomato plants against *Tomato spotted wilt virus* and *Potato virus Y*

**DOI:** 10.1038/s41598-018-28677-3

**Published:** 2018-07-09

**Authors:** Despoina Beris, Ioannis Theologidis, Nicholas Skandalis, Nikon Vassilakos

**Affiliations:** 1 Foundation for Research and Technology, Institute of Molecular Biology and Biotechnology, Heraklion/Crete, GR-71110 Greece; 2 Present Address: Keck School of Medicine, University of South California, 2020 ZONAL AVE. Off Campus, Los Angeles, USA

## Abstract

Plant growth promoting rhizobacteria have been proposed as effective biocontrol agents against several fungal and bacterial plant pathogens. However, there is limited knowledge regarding their effect against viruses. In this study, *Bacillus amyloliquefaciens* strain MBI600 (MBI600), active ingredient of the biological fungicide Serifel® (BASF SE), was tested for its antiviral action in tomato plants. Drench, foliar or soil amendment applications of MBI600 reduced up to 80% the incidence of *Tomato spotted wilt virus* under two different sets of environmental conditions. In addition, drench application of MBI600 delayed *Potato virus Y* systemic accumulation. Transcriptional analysis of a range of genes associated with salicylic acid (SA)- or jasmonic acid - related defense, priming or basal defense against viruses, revealed the induction of the SA signaling pathway in tomato after MBI600 treatment, and discrete gene expression patterns in plant response to TSWV and PVY infection.

## Introduction

Viruses are among the most important plant pathogens as near half of the emerging epidemics have a viral etiology resulting in great agronomic losses worldwide^1^. *Tomato spotted wilt virus* (TSWV) and *Potato virus Y* (PVY) are enlisted in the top 10 plant viruses in terms of both economical and biological impact^2^. TSWV is the type member of the *Tospovirus* genus of the *Bunyaviridae* family, and has a genome consisting of one negative and two ambisense single-stranded RNA segments. TSWV is worldwide distributed infecting more than 800 plant species and is transmitted through thrips-vectors in a circulative propagative manner^2,3^. PVY (type member of genus *Potyvirus*, *Potyviridae* family) has a 9.7 kb positive single-stranded RNA genome, infects a wide range of plant species mainly in the Solanaceae family and is transmitted in nature through more than 40 aphid species in a non-persistent manner. In the Mediterranean basin, TSWV is a major pathogen for Solanaceae cultivation causing severe losses especially on tomato, one of the most important crops for the Mediterranean agricultural economy. In addition, PVY infection decreases yield and quality in potato, tomato, pepper and tobacco crops^2,4^.

Plants have evolved a sophisticated response network for the repulsion/avoidance or restriction of pathogens at the infection sites. The latter is known as systemic resistance and is described as an enhanced status of defense capacity in the non-exposed plant parts against various pathogens and insects^5,6^. Depending on the elicitor, systemic resistance can be categorized in two forms: (i) induced systemic resistance (ISR) activated by plant-growth promoting rhizobacteria (PGPR) and fungi (PGPF), and (ii) systemic acquired resistance (SAR) activated by a variety of pathogens or chemical compounds^6,7^. In both cases, systemic resistance is related to differential gene expression that is tightly linked to hormonal production^8^. Priming is considered as a distinct form of induced resistance caused by a chemical or biotic stimulus, which involves accumulation of latent signaling proteins into cells. In subsequent exposure to biotic or abiotic stress these dormant proteins are activated and initiate signal amplification resulting in a faster immune response^9^.

PGPR have been widely known for their ability to colonize plant roots and increase plant growth and yield through the uptake of nutrients and the production of growth factors and vitamins. In addition, PGPR can induce systemic resistance or act antagonistically to several soilborne phytopathogens due to the production of siderophores, bacteriosins and antibiotics^10^. Beside PGPR’s commercial use against fungal and bacterial diseases, there is an increasing number of studies regarding its potential action against viruses. *Bacillus* spp. have been reported to induce antiviral responses against *Cucumber mosaic virus* (CMV) in tomato^11^, pepper^12^ and arabidopsis^13^, and against PVY and *Potato virus X* (PVX) in potato plants^14^. Moreover, *Pseudomonas* spp. reduced disease severity of TSWV^15^ and *Tomato mottle virus* (ToMoV)^16^ in tomato and of *Banana bunchy top virus* (BBTV)^17^ in banana plants. The defense mechanism triggered by PGPR against viruses depends on the complicated interactions among PGPR, host plant and virus, involving mostly the salicylate (SA) signaling pathway and in some cases both SA and jasmonate (JA) pathways^13,18,19^.

Thus far, therapeutic measures for plant virus diseases are not available and their control is mainly based on prevention through the control of their insect vectors and the use of genetically resistant cultivars. However, the latter is not always feasible while the effective control of vectors often has huge environmental and economic impact. Therefore, it is imperative to identify new antiviral agents, either chemical or biological. In this study, the commercially used biological agent *Bacillus amyloliquefaciens* strain MBI600 (MBI600) (bio-fungicide Serifel®, BASF SE) was tested for its antiviral action against TSWV and PVY in tomato. The application of MBI600 significantly reduced TSWV incidence, and delayed PVY accumulation in the non-inoculated leaves of the tomato plants. The expression analysis of six defense related genes prior to and following virus inoculations, revealed the induction of a salicylic acid dependent resistance mechanism by MBI600, and a differential effect of the two viruses in plant gene expression.

## Results

### MBI600 application schemes reduced TSWV incidence in tomato plants under greenhouse and plant growth chamber conditions

**Figure 1:**
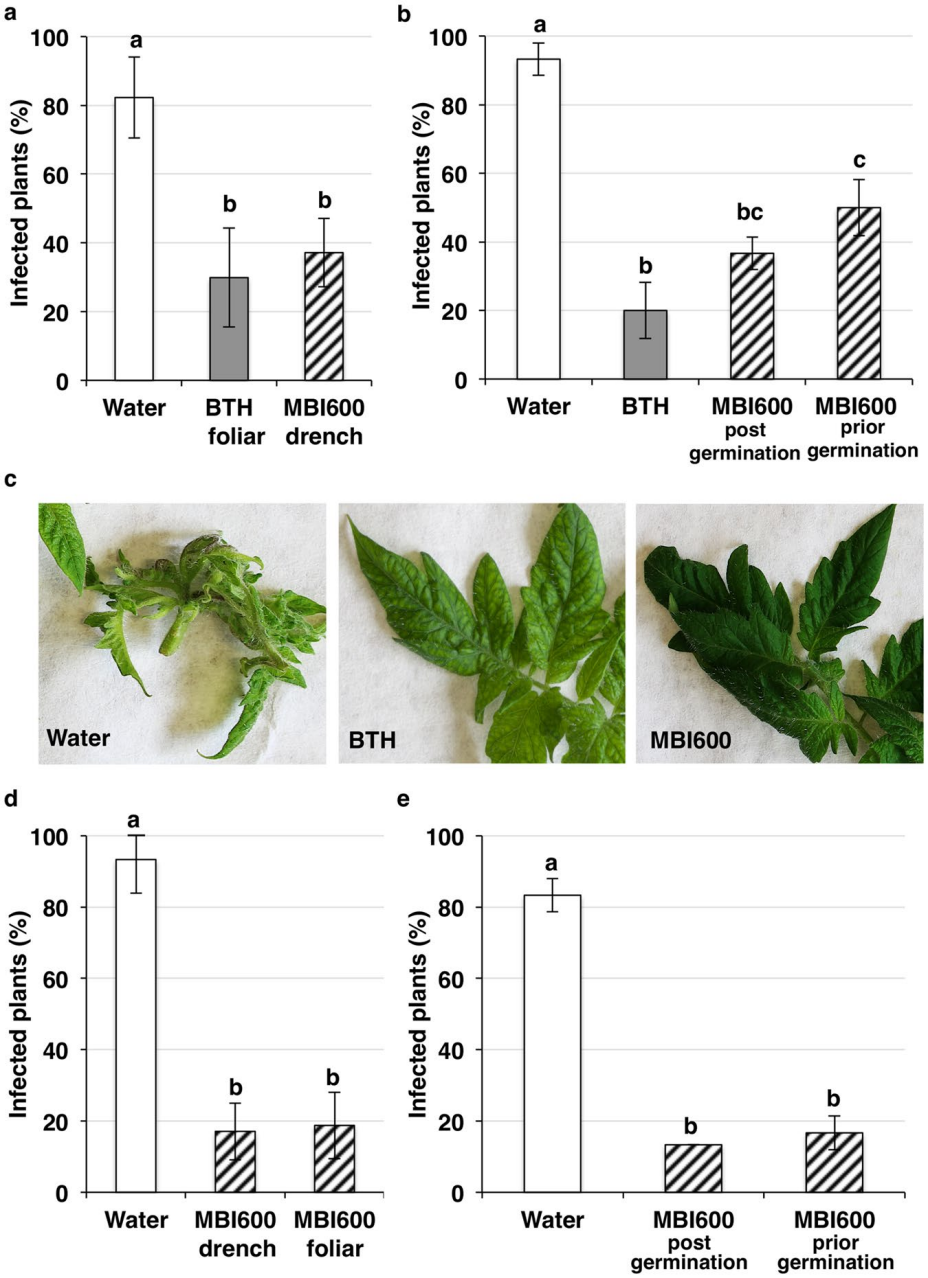
Effect of MBI600 in *Tomato spotted wilt virus* (TSWV) incidence. (**a**) Final percentage (%) of TSWV infected plants at 30 days post inoculation (dpi) after application of MBI600 compared to water treatment. (**b**) Percentage of plants detected TSWV-positive after treatment with water, BTH and MBI600 under greenhouse conditions. MBI600 was applied either in a triple-drench application program (post germination) or as a single soil amendment during sowing (prior germination). (**c**) Representative symptoms of TSWV in water treated plants (left picture) in contrast to the majority of the BTH and MBI600 treated plants, which were symptomless. (**d**) Percentage of plants infected with TSWV after water, MBI600 drench and foliar application under growth chamber conditions. (**e**) Percentage of TSWV incidence in plants treated with MBI600 in post and prior germination application schemes compared to water treatment under growth chamber conditions. Bars represent the mean value (± standard error) of (**a**) four and (**b**) two independent experiments conducted under greenhouse and (**d**) six and three (**e**) independent experiments under growth chamber conditions. In all cases, different letters indicate significant Tukey post’hoc differences between treatments (P < 0.05).

Under greenhouse conditions, a significant reduction of TSWV incidence was recorded in plants treated with either of MBI600 in a triple drench application scheme, or benzol [1,2,3] thiadiazol-7-carbothioic acid-S methyl ester (BTH), compared to the water (P < 0.0001 in all cases) but not between MBI600 and BTH treatments (P > 0.05) (Fig. 1a). Similarly to MBI600 triple drench application, the MBI600 single drench application in air-dried potting medium during sowing of tomato seeds (prior germination) resulted in a significant reduction of TSWV incidence compared to the water treatment (P < 0.0001 in all cases). However, in the MBI600 single application prior germination treatment a higher number of plants was found infected compared to the BTH (P = 0.0826) (Fig. 1b).

Representative tomato plants treated with each of water, BTH or MBI600 at 30 days post inoculation (dpi) with TSWV are illustrated in Fig. 1c and Supplementary Fig. S1. Plants treated with water showed severe TSWV symptoms including dark brown spots in the newly developed leaves, stunting and chlorosis (30/30 in total). In agreement with the ELISA results, only four out of 30 BTH-treated and seven out of 30 MBI600-treated plants exhibited symptoms. However, in both treatments, plants found positive for TSWV showed no difference in symptom severity compared to those treated with water.

The experiments conducted in the plant growth chamber showed that all three application methods of MBI600, resulted in reduced number of TSWV infected plants compared to control (water). Significant differences were recorded between MBI600 and the water (P < 0.0001 for all application methods), but not among MBI600 triple drench application and the other two MBI600 applications tested (foliar and dry-potting medium amendment) (P > 0.05) (Fig. 1d,e).

Importantly, in plants of MBI600 or BTH treatments tested TSWV negative in the apical leaves by ELISA, the virus was neither detected in the challenge-inoculated leaves at all time points examined. Moreover, the number of infected plants remained unaltered among the first and the following time points.

### MBI600 effect on PVY systemic accumulation and plant fresh and dry weight

**Figure 2:**
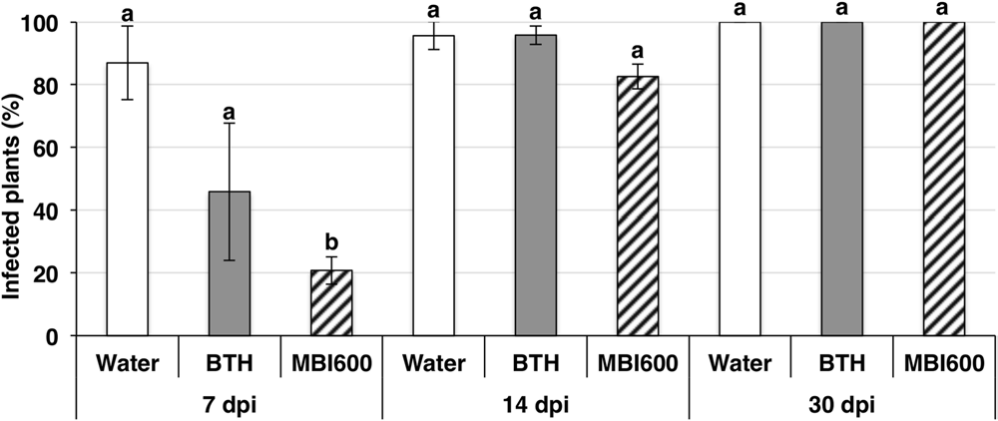
Effect of MBI600 on the progress of *Potato virus Y* (PVY) systemic infection. Percentage (%) of plants found positive with ELISA analysis at different time-points. Bars represent the mean value of three combined experiments (± standard error). Different letters represent statistically different data points at (P < 0.05) according to Tukey post’hoc comparisons. Ordinary least-square estimates are shown; dpi: days post inoculation.

ELISA analysis in systemic leaves showed a significant difference in PVY presence in MBI600 treated plants at seven dpi compared to BTH and water (P < 0.005 in all cases) but not among the three treatments at 14 and 30 dpi (Fig. 2). The fresh weight as well as the height of the MBI600 treated tomato plants were significant greater than those of the other two treatments at 30 dpi (Supplementary Fig. S2). In accordance with the results obtained from the ELISA analysis, mosaic symptom development in MBI600 and BTH treated plants was delayed approximately five days compared to those of the water treatment. First appearance of symptoms in water treatment occurred between 10–14 dpi and similar severity among the three treatments was observed at 30 dpi.

### Induction of the SA signaling pathway by MBI600 and diverse gene expression patterns in plant response to TSWV and PVY infection

To elucidate the molecular basis of MBI600 action, expression analysis of defense related genes was performed in MBI600 treated plants, just prior (time point 0) and at different time points post virus inoculations, and was compared to water treated plants (control) and BTH treatment. Genes were selected on the basis of their association with main plant defense responses against viruses, namely SA-based defense [*Non expresser of PR genes* (*SlNPR1*)^20^, *Pathogenesis related protein 1b* (*SlPR1b*.*1*)^21^, *RNA-dependent RNA polymerase 1* (*SlRdR1*)^22^], JA-based defense [*Lipoxygenase D* (*SlLoxD*)^23^ and *Coronatine insensitive 1* (*SlCOI1*)^24^], priming [*SlNPR1*^25^, *Mitogen-activated Protein Kinase 3* (*SlMPK3*)^26^] and RNAi basal defense against viruses (*SlRdR1*)^22^. P-values for all comparisons are provided in Supplementary Table S1.

**Figure 3:**
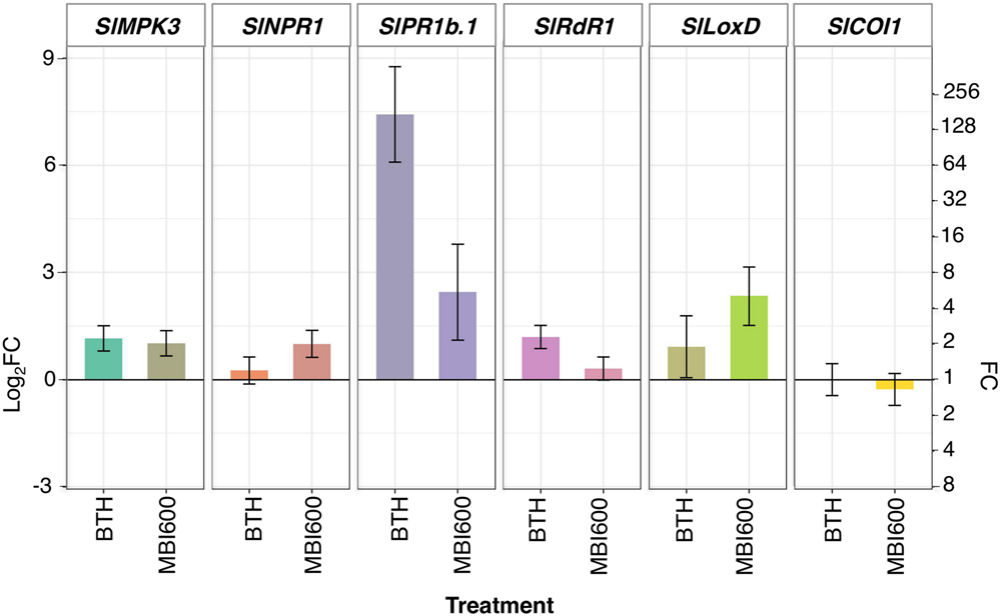
Effect of MBI600 on the transcriptional levels of selected defense genes (*SlMPK3*, *SlNPR1*, *SlPR1b*.*b*, *SlRdR1*, *SlLoxD* and *SlCOI1*) prior viral inoculation. Fold change (FC) represents the relative difference in expression between each treatment and water. Pools of four to five individual plants were analyzed and the results of two combined experiments are presented. *UBI3* was used as reference gene. Bars indicate the 95% confidence interval (2*SE). Comparisons whose confidence interval include the value 0 (1 in fold-change scale) are not significant at α = 5%.

Analysis of time point 0 performed for each of TSWV and PVY time courses, showed that plants treated with MBI600 and BTH exhibited elevated transcriptional levels of *SlMPK3* and *SlPR1b*.*1* in comparison to those treated with water (Fig. 3). Moreover, a significant increase of *SlNPR1* and *SlLoxD* transcripts was recorded in MBI600 treatment, and of *SlRdR1* transcripts in BTH treatment (Fig. 3).

Gene expression analysis following virus inoculations targeted the early stages of virus infection and was adjusted to the distinct response of MBI600-treated plants to each of the examined viruses, i.e. absence of local infection for TSWV or delay of systemic infection for PVY. Hence, in TSWV-challenged plants, analysis was performed in both inoculated and apical leaves at one and five dpi. In PVY-challenged plants, analysis was focused on apical leaves and conducted at one, two and five dpi while inoculated leaves were examined only at one dpi; an extra time point (two dpi) was added as PVY invades tomato plants systemically faster that TSWV (first detection of virus in apical leaves by ELISA, at approximately seven dpi for PVY, and 12 dpi for TSWV).

The analysis of TSWV-challenged leaves at one dpi showed that TSWV inoculation (water) resulted in the induction of *SlRdR1*, *SlLoxD* and *SlCOI1* compared to plants rub-inoculated solely with buffer (mock), whereas MBI600 and BTH treatments resulted in elevated levels of *SlNPR1* transcripts compared to both water and mock (Supplementary Fig. S3a). At five dpi, in MBI600 treatment *SlMPK3* and *SlNPR1* transcripts were higher than those of water and mock, whereas *SlLoxD* expression in all treatments was lower than mock (Supplementary Fig. S3a). The analysis of PVY-challenged leaves at one dpi showed a significant decrease of *SlPR1b*.*1* in both MBI600 and BTH treatments compared to water and mock, increased levels of *SlLoxD* in water compared to the rest treatments, and no differences among treatments for the rest of the examined genes (Supplementary Fig. S3b).

**Figure 4:**
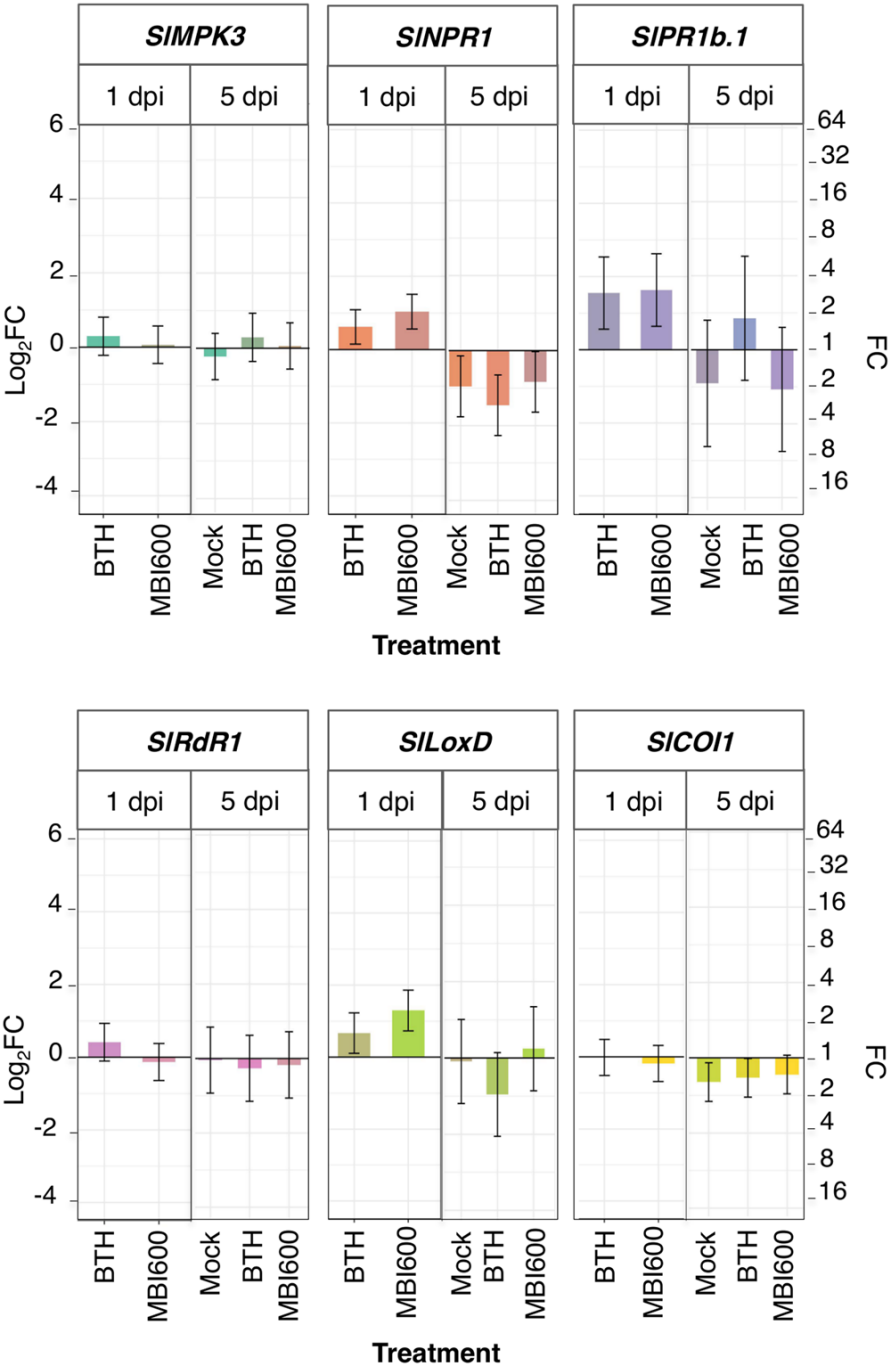
Effect of MBI600 on the transcriptional levels of the selected defense genes in the apical, non-inoculated (systemic) leaves at different time-points post *Tomato spotted wilt virus* inoculation. Five individual plants were analyzed with RT-qPCR. Mock demonstrates the expression of plants rub-inoculated solely with buffer. Fold change (FC) represents the relative difference in expression between each treatment and water. *UBI3* was used as reference gene. Bars indicate the 95% confidence interval (2*SE). Comparisons whose confidence interval include the value 0 (1 in fold-change scale) are not significant at α = 5% (FC: fold change, dpi: days post inoculation). At the time point of one dpi the mock treatment was not examined.

Expression analysis of apical leaves at one dpi, in TSWV challenge-inoculated plants showed elevated *SlNPR1*, *SlPR1b*.*1* and *SlLoxD* expression in MBI600 and BTH treatments compared to water. At five dpi, in mock, MBI600 and BTH treatments *SlNPR1* and *SlCOI1* transcripts were lower than those of water treatment. No significant differences were recorded among treatments in the expression levels for the rest of the genes (Fig. 4).

**Figure 5:**
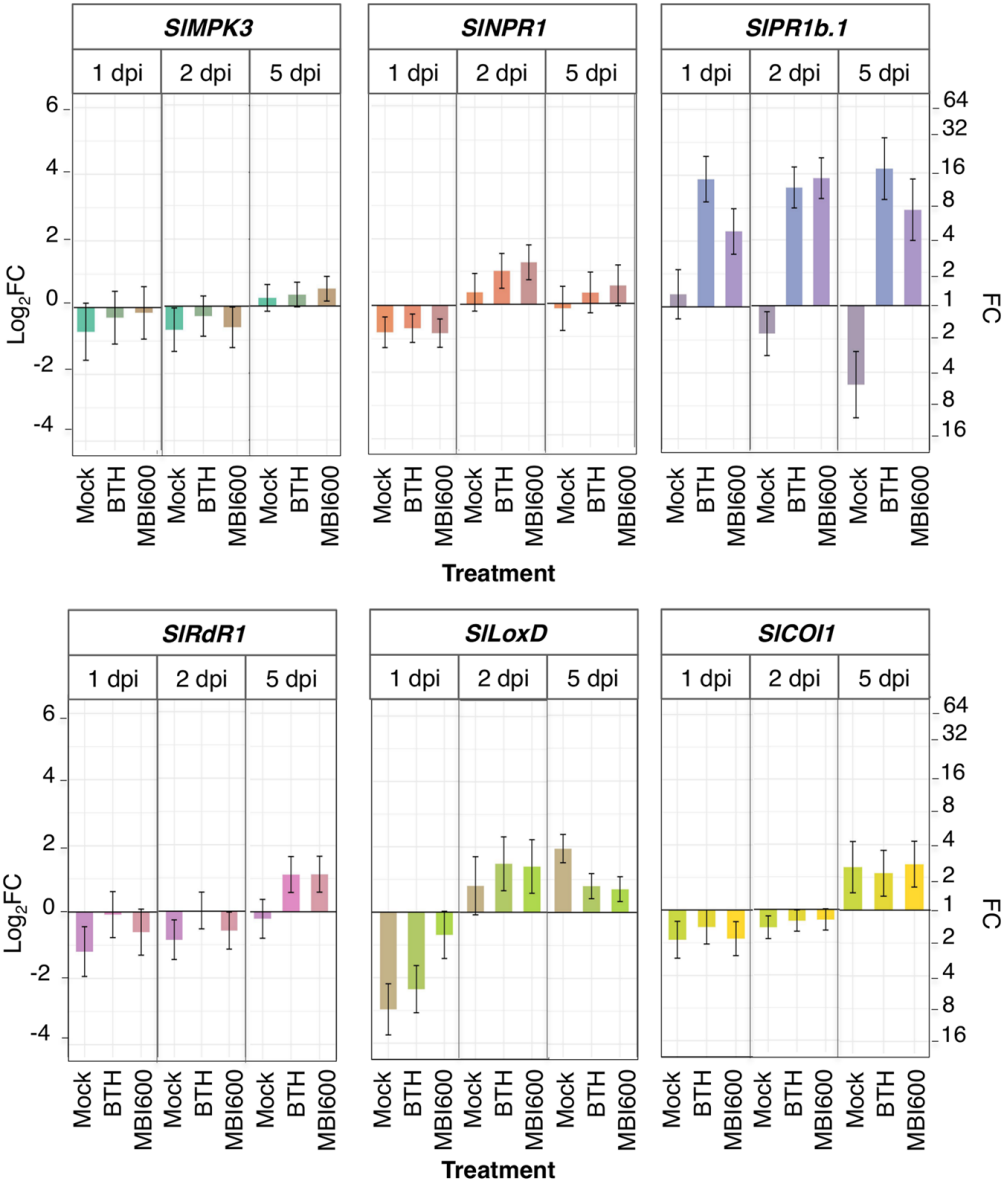
Effect of MBI600 on the transcriptional levels of the selected defense genes in the apical, non-inoculated (systemic) leaves at different time-points post *Potato virus Y* inoculation. Four individual plants were analyzed with RT-qPCR. Mock demonstrates the expression of plants rub-inoculated solely with buffer. Fold change (FC) represent the relative difference in expression between each treatment and water. *UBI3* was used as reference gene. Bars indicate the 95% confidence interval (2*SE). Comparisons whose confidence interval include the value 0 (1 in fold-change scale) are not significant at α = 5% (FC: fold change, dpi: days post inoculation).

In PVY-inoculated plants, analysis of systemic leaves revealed a more complex expression profile for most genes (Fig. 5). *SlMPK3* transcripts showed a slight increase in MBI600 treatment compared to water at five dpi. At all examined time points, transcriptional levels of *SlNPR1* or *SlLoxD* were similar among mock, MBI600 and BTH treatments, but were altered in time compared to water. *SlPR1b* transcripts in MBI600 and BTH treatments were higher than water and mock at all time points whereas *SlPR1b* levels were higher in water compared to mock, at two and five dpi. *SlRdR1* transcripts were increased significantly in BTH and MBI600 treatments compared to water and mock at five dpi. Finally, *SlCOI1* expression in mock, BTH and MBI600 treatments was higher than that of water treatment, at five dpi (Fig. 5).

To verify consistency among experiments in plant response to the applied treatments, plants were also tested for virus infection. TSWV challenged plants sampled at five dpi were left to further grow and tested at 30 dpi with ELISA. All water treated plants (5/5) were found positive for TSWV, whereas TSWV was detected in only one out of five plants of each MBI600 and BTH treatments. Similarly, in PVY experiment plants sampled at two dpi were tested with ELISA at 30 dpi and the virus was detected in only one out four plants of water treatment and in none of the other two treatments. Moreover, systemic virus accumulation was examined in parallel to gene expression by RT-qPCR in plants sampled at five dpi. PVY was detected in four out of four plants of water treatment, and in low quantity in three out of four plants of MBI600 treatment and four out of four plants of BTH treatment (Supplementary Table S2). For both experiments, plants of one dpi were not tested as the inoculated leaf was detached too early, rendering unlikely the occurrence of virus systemic movement.

## Discussion

Beneficial bacteria in the rhizosphere (PGPR) have been used for years as environmental-friendly biocontrol agents against fungal and bacterial diseases^27,28^ but thus far not against plant viruses. In this study, the antiviral potential of *Bacillus amyloliquefaciens* strain MBI600, active ingredient of the biological fungicide Serifel® (BASF SE), was tested in tomato. Distinguishing from previous PGPR studies^15^, a significant reduction, from 50% to 80%, of TSWV incidence was recorded in the various MBI600 application schemes tested (Fig. 1). The antiviral effect of MBI600 was proved robust under both plant growth chamber and greenhouse conditions although higher disease reduction scores were achieved under the former, presumably due to a better physiological state of the plants. Moreover, single dry-potting medium amendment prior germination and foliar applications performed equally well to triple drench application against TSWV, offering flexibility in a potential incorporation of MBI600 in integrated pest management programs. MBI600 application against PVY resulted in reduced virus accumulation at the early stages of the infection and delay of virus detection in apical leaves. Salicylic acid analogues like BTH were shown to induce resistance against several viruses including TSWV^29,30^ and PVY^31^. Interestingly, the effect of MBI600 against the two viruses resembled that of BTH. The later, in agreement with previous publications, reduced significantly TSWV incidence and delayed systemic movement of PVY in tomato plants. However, in the PVY experiment and in contrast to MBI600, BTH had no effect on plant growth in terms of fresh and dry weight of treated plants that had similar or inferior height to those of the control. The negative effect of SA synthetic chemicals on plant development that is commonly observed, especially in the absence of plant pathogen infection, consists a drawback for its wide adoption in the agricultural practice^32^.

Hormonal defense responses of plants against viruses are based mainly on SA and secondarily on JA^8^. SA is crucial for local and systemic resistance, through its involvement in (R)-gene resistance, basal immune responses and the connection between the SA-based defense and the RNA silencing antiviral mechanism^8,33,34^. SA-based defense response includes the activation of the mitogen activated kinase cascade that leads to the up-regulation of *NPR1*, which in turn induces the transcription of pathogenesis related genes (*PRs*)^19,20,25,26,35^. Moreover, treatment with SA or biologically active SA analogs induced *NtRDRP1* expression. The same gene is induced after infection with several viruses including PVY, and is considered as a basic component of plant defense against virus infection^22,36^. Furthermore, the crucial role of SA in plant resistance against both TSWV and PVY has been demonstrated in several studies^31,34,37,38^.

Gene expression analysis that was performed at time point 0 (Fig. 3) corresponded to seven days after the first and one day after the second MBI600 or BTH applications. In line with the literature, BTH treatment resulted in increased transcripts of *SlMPK3*, *SlPR1b*.*1* and *SlRdR1* denoting a primed state of plants or induction of SAR^20,26,29,39^. A simultaneous recording of low *SlNPR1* transcripts, similar to those of the water treatment, could be attributed to regulatory factors associated with the high expression of *SlPR1b*.*1*^40^. Likewise, the elevated *SlMPK3*, *SlNPR1* and *SlPR1b*.*1* transcripts recorded in MBI600 treated plants, suggested the induction of a SA-dependent response or priming^26,41^. However, the results from the expression analysis of the two genes related to JA-dependent response were inconclusive; the elevated *SlLoxD* transcripts in MBI600 treated plants indicated a possible parallel activation of the JA-dependent signaling pathway by MBI600^41,42^, hypothesis not confirmed by the expression levels of *SlCOI1*^24,43^, which were similar to the water treatment. Therefore, further experiments are required in order to clarify the role of JA-signaling in MBI600 action. Notably, in a rising number of studies, PGPR induced plant defense by activation of both SA and JA/ET signaling pathways, whereas the SA production in growth medium by *Bacillus* spp. has also been reported^19,41,44,45^.

The hypothesis that MBI600 activates the SA-dependent signaling pathway was further supported by the similarity in gene expression profiles between MBI600 and BTH treated plants after challenge with viruses. In MBI600 treated plants challenged with TSWV, occurrence of a priming status at five dpi was indicated by the elevated transcriptional levels of *SlMPK3* and *SlNPR1* in the inoculated leaves. Except for *SlLoxD*, the rest of the genes were expressed similarly to the mock treatment, probably reflecting the absence of infection. Correspondingly, in apical leaves after an increase of SA-associated genes in MBI600 treatment at one dpi, transcripts of most genes were similar among treatments at five dpi. The higher *SlNPR1* transcripts in water treatment are possibly associated with plant’s response to the initiation of TSWV systemic infection. Moreover, in plants of the water treatment the analysis of inoculated leaves at one dpi showed an initial increase in the expression of most genes examined, in comparison to mock-inoculated plants, which was diminished at five dpi. Intriguingly, *SlPR1b*.*1* levels were found similar or lower to mock, in contrast to a number of publications reporting increased *PR1* and SA levels in various hosts after TSWV infection^37,46,47^. However, in the latter studies gene expression analysis was performed at a later stage of virus infection, with a significant *SlPR1b*.*1* increase been recorded after seven dpi^46^. Therefore, it is possible that the increased *SlLoxD* and *SlCOI1* transcripts, recorded at one dpi at the site of inoculation, indicated an initial induction of JA pathway by TSWV in the expense of the SA one, so that a successful infection would be established. At a later stage, the above condition was reversed so that attractiveness to thrips-vectors would be favored, in the frame of TSWV’s manipulation of the SA-JA cross talk of plants^46^. Thus, it is plausible that the early activation of SA by MBI600 disrupted the modulation of host defense by TSWV, leading to high levels of resistance.

PVY infection has been also shown to interfere with the SA and JA defense signaling in potato^34,38,48,49^. In this study, a complex pattern in the transcriptional level of most genes examined was revealed at the early stages of PVY infection, which was changed in time. The perturbations especially in the expression of *SlPR1b*.*1*, *SlLoxD* and *SlCOI1* in water treated plants may indicate a switch between SA and JA signaling at five dpi. MBI600 application altered the expression profile of PVY infected plants with most pronounced differences been recorded in transcripts of *SlPR1b*.*1*, *SlRdR1* and *SlLoxD*, at the same time point of five dpi. The degree that these alterations are related with the resistance induced by MBI600 in tomato against PVY remains to be further investigated. However, similarly to the results of the present study, in the PVY-potato pathosystem, SA affected PVY replication in the inoculated leaves without preventing systemic movement of the virus, whereas it played an important role in the inhibition of PVY movement in parenchymal tissue, but not in vascular veins^34,38^. Most recently, it was shown that the HCPro of *Turnip mosaic virus* (TuMV) has the ability to negatively control the SA pathway in arabidopsis, through the interaction with, and negative regulation of the SA-binding protein AtCA1^50^. Surprisingly, a reduction of *SlPR1b*.*1* transcripts was recorded in the inoculated leaves of MBI600 treated plants in contrast to apical leaves, at one dpi. It is possible that the cumulative activation of the SA pathway by both MBI600 treatment and PVY infection resulted in its negative regulation at the site of infection, equivalently to the activation of a negative SA feedback loop proposed for arabidopsis plants overexpressing the SA-binding protein AtCA1, and infected with TuMV^50^. Alternatively, *PR1b* expression in inoculated leaves has been negatively related to resistance in potato, being higher in symptomatic than asymptomatic genotypes, whereas it was similar between genotypes in apical leaves^38^.

Overall, both the exhibited phenotype and gene expression analysis of MBI600 treated plants suggested the induction of a resistance mechanism against TSWV and PVY which is compatible with the model of the enhancement of antiviral resistance of susceptible plants after exogenous application of SA. Although several aspects of this model remain to be clarified, there are evidence suggesting that SA enhances the RNA-silencing antiviral defense of plants through the induction of *RdR1* and the triggering of several redundant pathways, probably in a dicer-like protein independent manner (reviewed in^8^). It is evident that there is a combination of pathways that leads to resistance or disease which is unique for every host-virus species combination due to the specific interaction and compatibility of each virus with host components and the counter measures each virus possesses against plant defenses^1^. The results presented in this study demonstrated that the biological fungicide *B*. *amyloliquefaciens* MBI600 could have an additional, although not uniform, antiviral effect. As the timing and intensity of response are extremely important for the outcome of a plant-virus interaction more experiments are required involving additional host-virus combinations and verification in the field. Nonetheless, considering the limited means for the control of viral diseases in the field, the capacity for a secondary function against viruses of a registered, environmentally friendly fungicide is essential in both economic and environmental terms.

## Methods

### Plants, virus isolates and Bacillus strain

Throughout this study, *Solanum lycopersicum* ‘Belladonna F1′ (Hazera) plants were used. Viruses were: (i) TSWV isolates, TSWV-619, TSWV-1729 and TSWV-4287 belonging to the same phylogenetic group, which originated from two pepper and one tomato plants, respectively, (ii) PVY isolates, PVY-1 and PVY-4122, derived from potato and tomato, respectively. TSWV was maintained in *Nicotiana rustica* and PVY in *N*. *tabacum* cv. ‘Samsun’. *Bacillus amyloliquefaciens* strain MBI600 (MBI600) was provided by BASF SE in the form of endospores.

### Plant growth conditions

Experiments were conducted under two different sets of environmental conditions; the first in an insect-proof greenhouse with controlled environmental conditions and the second in a plant growth chamber (MLR-352H, Panasonic). Greenhouse conditions were temperature typically at 25 °C/20 °C (day/night) with occasionally recorded extreme values of 28 °C/17 °C (day/night), and photoperiod adjusted to 16 h-light with supplementary to daylight illumination provided by GreenPower LED flowering DR/W lamps (22 µmol/s). Experiments were carried-out from February to November for three consecutive years. Growth chamber conditions were set at 16h-light photoperiod with light intensity of 20000 lux, temperature 25 °C/22 °C (day/night) and relative humidity 85% (day) and 90% (night). Plants were grown in soil-less potting medium (Potgrond P, Klasmann) in pots with dimensions 90 mm × 90 mm × 100 mm. No additional fertilization was provided.

### MBI600, BTH application and virus inoculation

MBI600 viable spores were diluted in sterile water to the recommended by the company dosage for tomato plants (5.5 × 10^7^ cfu/ml). MBI600 was applied either as drenching (60 ml per pot) or foliar spray (spraying until run-off at a maximum rate estimated up to 15 ml of suspension per plant, depending on plant age). Preliminary experiments indicated the adoption of a triple, weekly drench or spray application program. The first application was carried out in tomato plants at the developmental stage of the second leaf on main shoot unfolded (phenological developmental stage BBCH 102). Virus (TSWV or PVY) challenge inoculations of the plants were performed one day after the second MBI600 application. Foliar application experiments of MBI600 were conducted solely in the growth chamber due to low relative humidity conditions (lower than 50%) in the greenhouse (use of air-conditioners for temperature control) which could compromise the viability of bacillus spores on leaf surface. Furthermore, a single drench application of MBI600 was tested in air-dried potting medium during sowing of tomato seeds (prior germination). In detail, 1,5 litre of 5.5 × 10^7^ cfu/ml suspension was mixed with 1 kg of dry potting medium and the resulting mixture was used for sowing of tomato seeds. Subsequently, plants were transplanted into the nine cm pots and virus challenge inoculation was carried-out at the developmental stage of the third leaf on main shoot unfolded (BBCH 103–104) similarly to triple drench or foliar application programs.

Benzol [1,2,3] thiadiazol-7-carbothioic acid-Smethyl ester, (BTH, Sigma), a chemical analogue of SA, was used as positive control for SAR activation. Chemical analogues of SA have been reported to induce resistance against TSWV^29^ and PVY^31^. In all experiments, regardless of the MBI600 application method, BTH was used at 100 mM in a triple foliar weekly application program identical to that of MBI600. Moreover, controls included triple weekly water treatment of virus challenged plants and mock inoculated plants.

Viral inocula were prepared from TSWV- or PVY-infected *Nicotiana* sp. leaf tissues, ground in 10 mM-sodium phosphate, 0.2% w/v DIECA buffer pH 7, in a 1:3 w/v dilution, containing 3% w/v active carbon and carborudum. Plants were rub- inoculated^4,29,31^ with TSWV or PVY inoculum, onto the youngest fully expanded composite leaf and kept for 24 hours in the dark at 22 °C. Inocula were also applied to local lesion hosts of TSWV and PVY (*Chenopodium quinoa* and *C*. *amaranticolor* respectively) to quantify the infectivity of each inoculum.

### Experimental design

MBI600 action against TSWV was examined in two sets of experiments: (a) in the greenhouse, testing the following treatment combinations: (i) water, BTH, MBI600 triple drench application (four independent experiments), and (ii) water, BTH, MBI600 single drench application in dry potting medium (two independent experiments). A randomized complete block design with three blocks was used. Each block consisted of plots with 10 plants per plot (30 plants per treatment). (b) In the plant growth chamber, testing two different combinations of MBI600 application: (i) water, MBI600 triple drench application, MBI600 triple foliar application; (ii) water, MBI600 triple drench application, MBI600 single drench application in dry potting medium. Three experiments were carried-out for the first and two for the second combination using 15 plants per treatment. Experiments examining MBI600 action against PVY were carried out only in the greenhouse following the experimental design of TSWV.

### Viral disease assessment

The progress of viral diseases was evaluated visually by symptom monitoring and by double-antibody sandwich enzyme-linked immunosorbent assay^51^ (DAS-ELISA) utilizing commercial antibodies (LOEWE Biochemica GmbH). ELISA was performed in both challenged inoculated and the apical non-inoculated leaves (to check systemic virus movement) at three different time points, 14, 19 and 30 dpi for TSWV, and 7, 14 and 30 dpi for PVY. Samples were considered positive when their A_405_ was higher than three times the mean A_405_ of three healthy control samples.

### Gene expression analysis

The effect of MBI600 on the transcript levels of selected tomato defense-related genes was examined just before and following challenge inoculations with each of TSWV and PVY in two separated time-course studies using quantitative RT-PCR (RT-qPCR). The defense-associated genes were *Mitogen-activated Protein Kinase 3* (*SlMPK3*), *Non expresser of PR genes* (*SlNPR1*), *Pathogenesis related protein 1b* (*SlPR1b*.*1*), *RNA-dependent RNA polymerase 1* (*SlRdR1*), *Lipoxygenase D* (*SlLoxD*) and *Coronatine insensitive 1* (*SlCOI1*). Transcript levels of the target genes were quantified relatively to ubiquitin using primers UBI3-F and UBI3-R^52^. Primers are described in Supplementary Table S2. Experiments were carried out in the plant growth chamber at the environmental settings already described. Plant treatments with each of MBI600, BTH and water, as well as virus challenge inoculations were performed as described previously in the triple drench MBI600 application program. Mock inoculated plants were also included. Gene expression was examined at 0, 1, 2 and 5 days post inoculation (dpi). Plants were sampled once. For time point 0, just before virus inoculations, leaves derived from up to five different plants per treatment were analyzed in pools (45 plants in total). For time points 1–5 dpi, four or five plants were individually analyzed per treatment. Moreover, the challenge inoculated and two apical young leaves were analyzed separately (local/apical). Total RNA was isolated from liquid nitrogen frozen samples using Nucleozol (Macherey-Nagel) according to manufacturer’s instructions. All subsequent steps for transcriptional level analysis and RT-qPCR were performed as described before^4^. In addition, plants challenge-inoculated with TSWV and sampled at five dpi, were left in the greenhouse and tested for systemic virus presence at 30 dpi by ELISA. Similarly, plants challenge-inoculated with PVY and sampled at two and five dpi, were tested for virus presence in apical leaves with ELISA and RT-qPCR respectively (Supplementary Table S3).

### Assessment of height, fresh and dry weight

In a PVY infectivity experiment, total height, from hypocotyl to shoot apical meristem, was measured in plants from water, BTH and MBI600 treatments (90 plants in total) at 30 dpi. Plants were subsequently separated in three pools of ten plants per treatment and the fresh weight was measured. The samples were then incubated in an oven for 48 hours at 60 °C until completely dried and then their dry weight was measured.

### Data statistical analysis

Disease incidence after the infection with PVY and TSWV was modeled with the implementation of generalized linear mixed models (GLMMs) in the programming language R^53^. Proportion of infected plants was the response variable, while treatment, time post inoculation and their interaction were the fixed factors of the model. Sample ID and block were the random factors and error distribution was set to binomial. *glmer* function was used for the modelling runs and *lsmeans* function was used for the estimation of post-hoc Tukey tests.

Quantitative PCR analysis was performed using the method described in^54^. Ct values of each factor combination were analysed in the context of a Linear Mixed Model (LMM) as dependent variables while treatments, genes and their interactions comprised the fixed factors. Samples nested within treatment and experiment were the random effects of the models. Log fold changes and confidence intervals of the relative expression of all genes were calculated by formulating the contrasts of the model estimates according to equation 5 of the report of^54^. Estimates of Ct values of the *UBI3* gene were used as reference. In cases where multiple plates were needed for the coverage of all samples, multi-run calibration was implemented with the use of replicated template samples across respective runs (see^55^ and references there in). After normalization, calibration samples were excluded from the analysis.

## Electronic supplementary material

Supplementary figures and tables

## References

[1] Lewsey, M., Palukaitis, P. & Carr, J. P. Plant–virus interactions: defence and counter-defence. In *Annual Plant Reviews Volume 34: Molecular Aspects of Plant Disease Resistance* (ed. Parker, J.) 134–176 (Wiley-Blackwell, 2008). doi:10.1002/9781444301441.ch6

[2] Scholthof K-BG (2011). Top 10 plant viruses in molecular plant pathology: Top 10 plant viruses. Mol. Plant Pathol..

[3] German TL, Ullman DE, Moyer JW (1992). Tospoviruses: diagnosis, molecular biology, phylogeny, and vector relationships. Annu. Rev. Phytopathol..

[4] Skandalis N (2016). Effect of pyraclostrobin application on viral and bacterial diseases of tomato. Plant Dis..

[5] Spoel SH, Dong X (2012). How do plants achieve immunity? Defence without specialized immune cells. Nat. Rev. Immunol..

[6] Fu ZQ, Dong X (2013). Systemic Acquired Resistance: Turning local infection into global defense. Annu. Rev. Plant Biol..

[7] Pieterse CMJ (2014). Induced systemic resistance by beneficial microbes. Annu. Rev. Phytopathol..

[8] Alazem M, Lin N-S (2015). Roles of plant hormones in the regulation of host–virus interactions. Mol. Plant Pathol..

[9] Conrath U (2011). Molecular aspects of defence priming. Trends Plant Sci..

[10] Beneduzi A, Ambrosini A, Passaglia LMP (2012). Plant growth-promoting rhizobacteria (PGPR): Their potential as antagonists and biocontrol agents. Genet. Mol. Biol..

[11] Zehnder GW, Yao C, Murphy JF, Sikora ER, Kloepper JW (2000). Induction of resistance in tomato against *Cucumber mosaic cucumovirus* by plant growth-promoting rhizobacteria. Biocontrol.

[12] Lee GH, Ryu C-M (2016). Spraying of leaf-colonizing *Bacillus amyloliquefaciens* protects pepper from *Cucumber mosaic virus*. Plant Dis..

[13] Ryu C-M, Murphy JF, Mysore KS, Kloepper JW (2004). Plant growth-promoting rhizobacteria systemically protect *Arabidopsis thaliana* against *Cucumber mosaic virus* by a salicylic acid and NPR1-independent and jasmonic acid-dependent signaling pathway. Plant J..

[14] Park K (2006). *Bacillus vallismortis* strain EXTN-1 mediated systemic resistance against *Potato virus Y* and *X* in the field. Plant Pathol. J..

[15] Kandan A (2005). Use of *Pseudomonas fluorescens*-based formulations for management of *Tomato spotted wilt virus* (TSWV) and enhanced yield in tomato. Biocontrol Sci. Technol..

[16] Murphy JF (2000). Plant growth-promoting rhizobacterial mediated protection in tomato against *Tomato mottle virus*. Plant Dis..

[17] Harish S, Kavino M, Kumar N, Balasubramanian P, Samiyappan R (2009). Induction of defense-related proteins by mixtures of plant growth promoting endophytic bacteria against *Banana bunchy top virus*. Biol. Control.

[18] Lee G, Lee S-H, Kim KM, Ryu C-M (2017). Foliar application of the leaf-colonizing yeast *Pseudozyma churashimaensis* elicits systemic defense of pepper against bacterial and viral pathogens. Sci. Rep..

[19] Gkizi D (2016). The Innate immune signaling system as a regulator of disease resistance and induced systemic resistance activity against *Verticillium dahliae*. Mol. Plant. Microbe Interact..

[20] Kohler A, Schwindling S, Conrath U (2002). Benzothiadiazole-induced priming for potentiated responses to pathogen infection, wounding, and infiltration of water into leaves requires the *NPR1*/*NIM1* gene in Arabidopsis. Plant Physiol..

[21] Block A, Schmelz E, O’Donnell PJ, Jones JB, Klee HJ (2005). Systemic acquired tolerance to virulent bacterial pathogens in tomato. Plant Physiol..

[22] Xie Z, Fan B, Chen C, Chen Z (2001). An important role of an inducible RNA-dependentRNA polymerase in plant antiviral defense. Proc. Natl. Acad. Sci..

[23] Hu T, Hu Z, Zeng H, Qv X, Chen G (2015). Tomato lipoxygenase D involved in the biosynthesis of jasmonic acid and tolerance to abiotic and biotic stress in tomato. Plant Biotechnol. Rep..

[24] Xie D-X, Feys BF, James S, Nieto-Rostro M, Turner JG (1998). COI1: An arabidopsis gene required for jasmonate-regulated defense and fertility. Science.

[25] Yi SY, Min SR, Kwon S-Y (2015). NPR1 is Instrumental in priming for the enhanced flg22-induced MPK3 and MPK6 activation. Plant Pathol. J..

[26] Beckers GJM (2009). Mitogen-Activated Protein Kinases 3 and 6 are required for full priming of stress responses in *Arabidopsis thaliana*. Plant Cell Online.

[27] Chen Y (2013). Biocontrol of tomato wilt disease by *Bacillus subtilis* isolates from natural environments depends on conserved genes mediating biofilm formation. Environ. Microbiol..

[28] Romero FM, Marina M, Pieckenstain FL (2016). Novel components of leaf bacterial communities of field-grown tomato plants and their potential for plant growth promotion and biocontrol of tomato diseases. Res. Microbiol..

[29] Mandal B (2008). Biological and molecular analyses of the acibenzolar-S-methyl-induced systemic acquired resistance in flue-cured tobacco against *Tomato spotted wilt virus*. Phytopathology.

[30] Pappu HR, Csinos AS, McPherson RM, Jones DC, Stephenson MG (2000). Effect of acibenzolar-S-methyl and imidacloprid on suppression of tomato spotted wilt Tospovirus in flue-cured tobacco. Crop Prot..

[31] Nie X (2006). Salicylic acid suppresses *Potato virus Y* isolate N: O-induced symptoms in tobacco plants. Phytopathology.

[32] Sun T-J (2015). A novel pyrimidin-like plant activator stimulates plant disease resistance and promotes growth. PLOS ONE.

[33] Alamillo JM, Saénz P, García JA (2006). Salicylic acid-mediated and RNA-silencing defense mechanisms cooperate in the restriction of systemic spread of *Plum pox virus* in tobacco. Plant J..

[34] Baebler Š (2014). Salicylic acid is an indispensable component of the Ny-1 resistance-gene-mediated response against *Potato virus Y* infection in potato. J. Exp. Bot..

[35] Tsuda K (2013). Dual regulation of gene expression mediated by extended MAPK activation and salicylic acid contributes to robust innate immunity in *Arabidopsis thaliana*. PLOS Genet..

[36] Rakhshandehroo F, Takeshita M, Squires J, Palukaitis P (2009). The influence of RNA-dependent RNA polymerase 1 on *Potato virus Y* infection and on other antiviral response genes. Mol. Plant. Microbe Interact..

[37] López-Gresa MP (2016). Salicylic acid is involved in the basal resistance of tomato plants to *Citrus exocortis viroid* and *Tomato spotted wilt virus*. PLOS ONE.

[38] Baebler Š (2011). Dynamics of responses in compatible potato - *Potato virus Y* interaction are modulated by salicylic acid. PLoS ONE.

[39] Yi H-S, Yang JW, Choi HK, Ghim S-Y, Ryu C-M (2012). Benzothiadiazole-elicited defense priming and systemic acquired resistance against bacterial and viral pathogens of pepper under field conditions. Plant Biotechnol. Rep..

[40] Mou Z, Fan W, Dong X (2003). Inducers of plant systemic acquired resistance regulate NPR1 function through redox changes. Cell.

[41] Abdallah AB (2017). R. *et al*. Involvement of lipopeptide antibiotics and chitinase genes and induction of host defense in suppression of Fusarium wilt by endophytic *Bacillus* spp. in tomato. Crop Prot..

[42] García-Gutiérrez L (2013). The antagonistic strain *Bacillus subtilis* UMAF6639 also confers protection to melon plants against cucurbit powdery mildew by activation of jasmonate- and salicylic acid-dependent defence responses: UMAF6639 elicits ISR via JA and SA signalling. Microb. Biotechnol..

[43] Katsir L, Chung HS, Koo AJ, Howe GA (2008). Jasmonate signaling: a conserved mechanism of hormone sensing. Curr. Opin. Plant Biol..

[44] van Wees SCM, de Swart EAM, van Pelt JA, van Loon LC, Pieterse CMJ (2000). Enhancement of induced disease resistance by simultaneous activation of salicylate- and jasmonate-dependent defense pathways in Arabidopsis thaliana. Proc. Natl. Acad. Sci. USA.

[45] Niu D-D (2011). The plant growth–promoting rhizobacterium *Bacillus cereus* AR156 induces systemic resistance in *Arabidopsis thaliana* by simultaneously activating salicylate- and jasmonate/ethylene-dependent signaling pathways. Mol. Plant. Microbe Interact..

[46] Abe H (2012). Antagonistic Plant defense system regulated by phytohormones assists interactions among vector insect, thrips and a Tospovirus. Plant Cell Physiol..

[47] Nachappa P, Margolies DC, Nechols JR, Whitfield AE, Rotenberg D (2013). *Tomato spotted wilt virus* benefits a non-vector arthropod, *Tetranychus urticae*, by modulating different plant responses in tomato. Plos ONE.

[48] Pompe-Novak M (2005). *Potato virus Y* induced changes in the gene expression of potato (Solanum tuberosum L.). Physiol. Mol. Plant Pathol..

[49] Petek M (2014). *Potato virus Y* infection hinders potato defence response and renders plants more vulnerable to Colorado potato beetle attack. Mol. Ecol..

[50] Poque S (2018). Potyviral gene-silencing suppressor HCPro interacts with Salicylic Acid (SA)-Binding Protein 3 to weaken SA-mediated defense responses. Mol. Plant. Microbe Interact..

[51] Clark MF, Adams AN (1977). Characteristics of the microplate method of enzyme-linked immunosorbent assay for the detection of plant viruses. J. Gen. Virol..

[52] Rotenberg D, Thompson TS, German TL, Willis DK (2006). Methods for effective real-time RT-PCR analysis of virus-induced gene silencing. J. Virol. Methods.

[53] R: a language and environment for statistical computing. Available at: https://www.gbif.org/tool/81287/r-a-language-and-environment-for-statistical-computing. (Accessed: 11th January 2018).

[54] Steibel JP, Poletto R, Coussens PM, Rosa GJM (2009). A powerful and flexible linear mixed model framework for the analysis of relative quantification RT-PCR data. Genomics.

[55] Ruijter JM, Ruiz Villalba A, Hellemans J, Untergasser A, van den Hoff MJB (2015). Removal of between-run variation in a multi-plate qPCR experiment. Biomol. Detect. Quantif..

