# Supplementary material for: Bacillus amyloliquefaciens strain MBI600 induces salicylic acid dependent resistance in tomato plants against Tomato spotted wilt virus and Potato virus Y
Source: Sci Rep. 2018 Jul 9;8:10320. doi: 10.1038/s41598-018-28677-3 (PMC6037670; doi:10.1038/s41598-018-28677-3)
Supplement: Supplementary file 1 — Supplementary figures and tables [file 41598_2018_28677_MOESM1_ESM.pdf]

Bacillus amyloliquefasciens **strain MBI600 induces salicylic acid dependent resistance in tomato plants against *Tomato spotted wilt virus* and *Potato virus Y***

Despoina Beris, Ioannis Theologidis, Nicholas Skandalis [#] and Nikon Vassilakos[*]

Foundation for Research and Technology, Institute of Molecular Biology and Biotechnology, Heraklion/Crete, GR-71110, Greece

# Present address: Keck School of Medicine, University of South California, 2020 ZONAL AVE. Off Campus, Los Angeles, USA

[*] n.vassilakos@bpi.gr

a

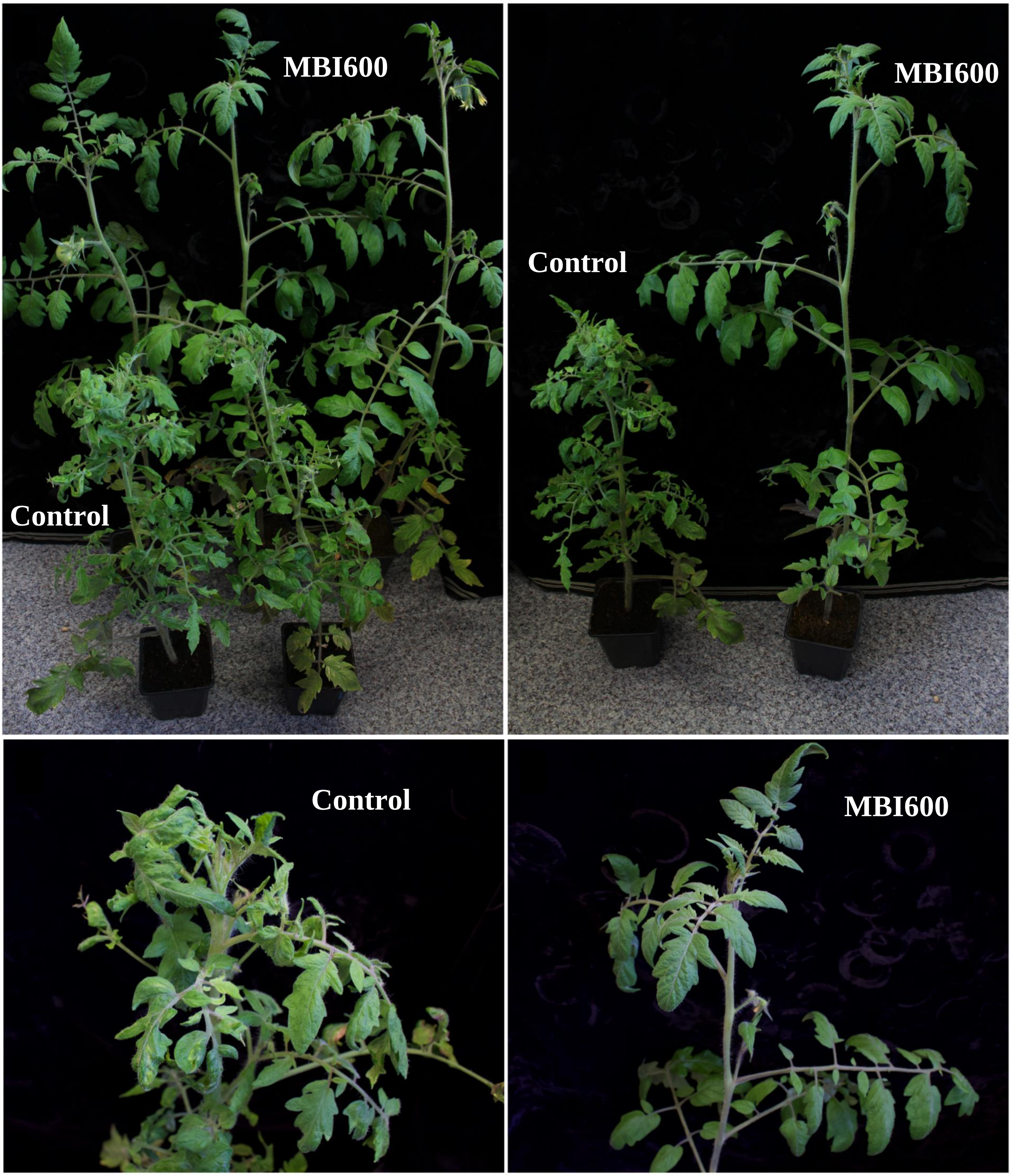

b

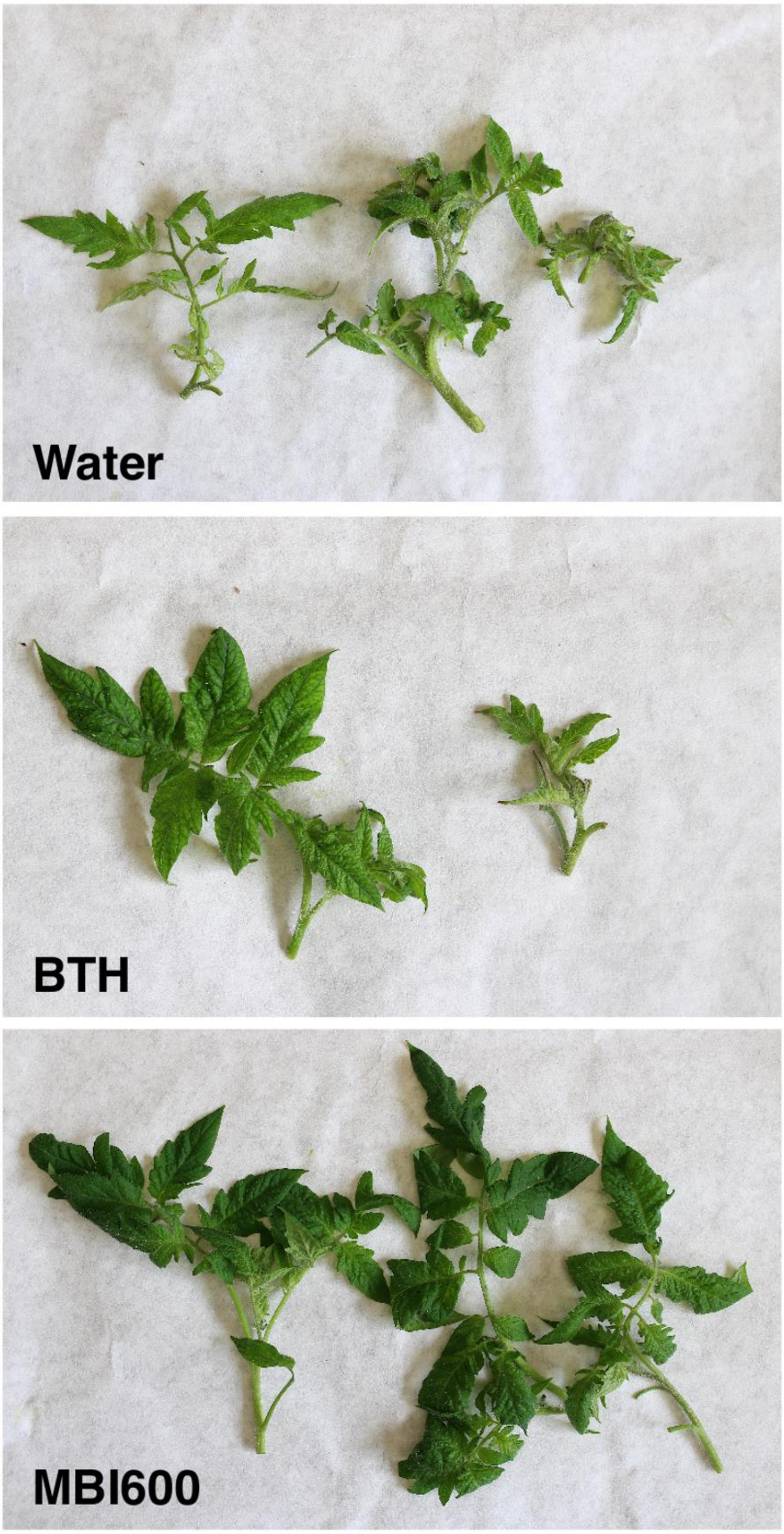

Supplementary Figure S1. (a) Water treated (control) and MBI600 treated tomato plants at 30 days post inoculation (dpi) with *Tomato spotted wilt virus* (TSWV); Control and MBI600 treated tomato plants are indicated accordingly. The majority of MBI600 treated plants were ELISA tested negative for TSWV, symptomless and higher. In contrast, most control plants were TSWV-positive and developed severe symptoms including dark brown spots in younger leaves, stunting and chlorosis. (b) Representative apical leaves of tomato plants at 30 dpi with TSWV treated with Water, BTH and MBI600. In BTH treatment the majority of plants were ELISA tested negative for TSWV and symptomless (leaves on the left), while TSWV infected plants exhibited symptoms similar to those observed in water treatment (leaf on the right).

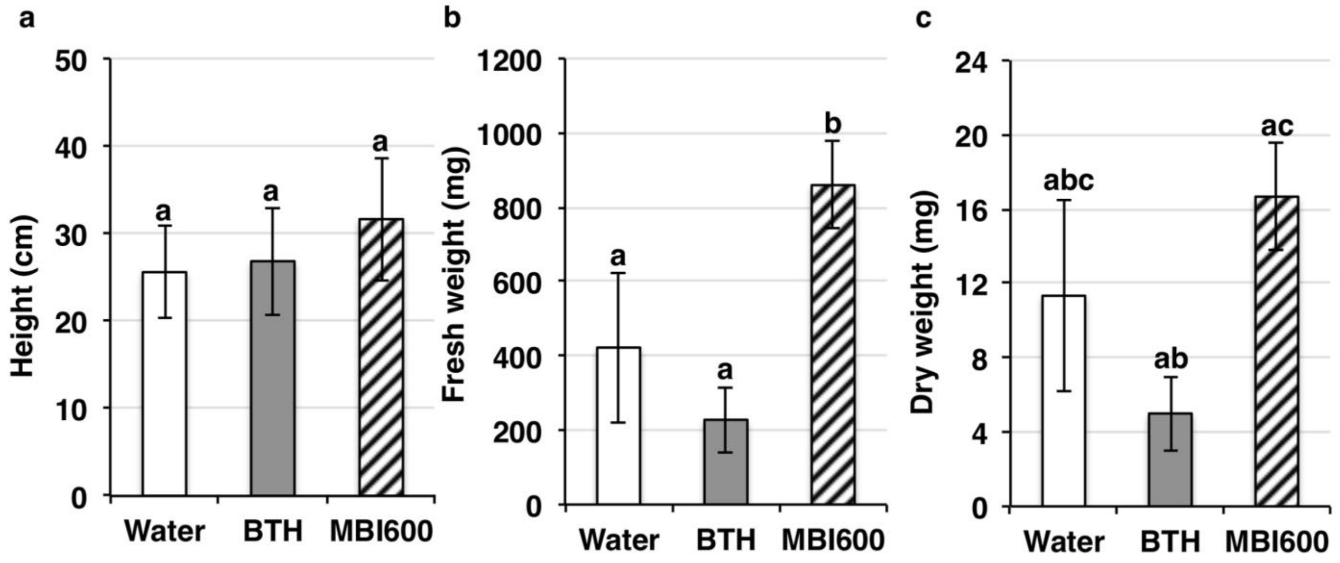

Supplementary Figure S2. Effect of MBI600 on height (a), fresh (b) and dry weight (c) of PVY-infected tomato plants. Three pools consisting of ten plants per treatment were analyzed separately. Bars represent the mean value of three biological pools. Different letters indicate statistically different values ($P<0.005$).

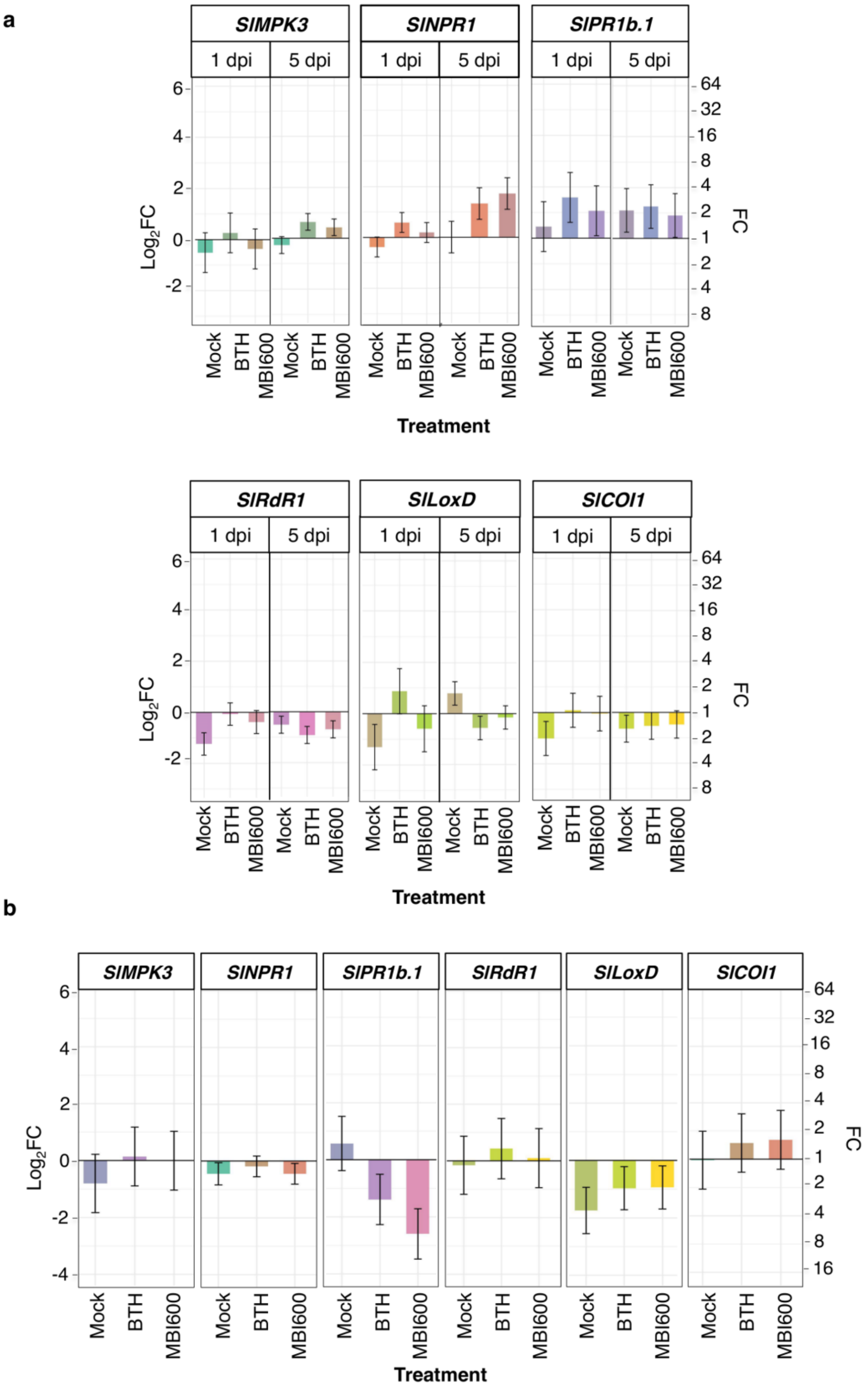

Supplementary Figure S3. Effect of MBI600 on the transcriptional levels of defense genes (*SlMPK3*, *SlNPR1*, *SlPR1b.b*, *SlRdR1*, *SlLoxD* and *SlCOI1*) in the (a) *Tomato spotted wilt virus* – (TSWV) challenge-inoculated leaves at one and five days post inoculation (dpi) and (b) *Potato virus Y* (PVY) inoculated leaves at one dpi. Four to five individual plants were analyzed separately with RT-qPCR. Fold change (FC) represent the relative difference in expression between each treatment and water. In both cases, mock demonstrates the expression of plants rub-inoculated solely with buffer. *UBI3* was used as reference gene. Bars indicate the 95% confidence interval (2*SE, n=5). Comparisons whose confidence interval include the value 0 (1 in fold-change scale) are not significant at α=5%.

	Gene	Contrast	estimate	SE	df	t.ratio	p.value
**Leaves prior viral inoculations [illegible]**	*SlMPK3*	Water-BTH	1,159801427	0,1774422	60,00	6,5360	<0,0001
		Water-MBI600	1,020637917	0,1774422	60,00	5,7520	<0,0001
	SlNPR1	Water-BTH	0,261379353	0,189423	60,00	1,3800	0,1727
		Water-MBI600	1,002188825	0,189423	60,00	5,2910	<0,0001
	SlPR1b.1	Water-BTH	7,422514846	0,6686223	60,00	11,1010	<0,0001
		Water-MBI600	2,44282511	0,6686223	60,00	3,6540	0,0005
	SlRdR1	Water-BTH	1,193334017	0,1619137	60,00	7,3700	<0,0001
		Water-MBI600	0,309813026	0,1619137	60,00	1,9130	0,0605
	SlLoxD	Water-BTH	0,920522906	0,4303845	57,18	2,1390	0,0367
		Water-MBI600	2,334376383	0,4076895	56,59	5,7260	<0,0001
	SlCOI1	Water-BTH	0,005196689	0,2230077	60,00	0,0230	0,9815
		Water-MBI600	-0,270680316	0,2230077	60,00	-1,2140	0,2296
**TSWV Inoculated leaves (1 dpi)**	*SlMPK3*	Water-Mock	-0,51442911	0,3899529	56,00	-1,3190	0,1925
		Water-BTH	0,25992029	0,3899529	56,00	0,6670	0,5078
		Water-MBI600	-0,36543234	0,3899529	56,00	-0,9370	0,3527
	SlNPR1	Water-Mock	-0,37918726	0,1962593	56,00	-1,9320	0,0584
		Water-BTH	0,58520673	0,1962593	56,00	2,9820	0,0042
		Water-MBI600	0,19122397	0,1962593	56,00	0,9740	0,3341
	SlPR1b.1	Water-Mock	0,44743675	0,4857128	56,00	0,9210	0,3609
		Water-BTH	1,58453192	0,4857128	56,00	3,2620	0,0019
		Water-MBI600	1,06758738	0,4857128	56,00	2,1980	0,0321
	SlRdR1	Water-Mock	-1,23670476	0,219551	55,05	-5,6330	<0,0001
		Water-BTH	-0,06809433	0,219551	55,05	-0,3100	0,7576
		Water-MBI600	-0,38175853	0,2233356	55,14	-1,7090	0,093
	SlLoxD	Water-Mock	-1,30407277	0,442264	54,42	-2,9490	0,0047
		Water-BTH	0,87084938	0,442264	54,42	1,9690	0,054
		Water-MBI600	-0,59099214	0,4494001	54,62	-1,3150	0,194
	SlCOI1	Water-Mock	-1,01591543	0,3313955	54,20	-3,0660	0,0034
		Water-BTH	0,08706204	0,3313955	54,20	0,2630	0,7938
		Water-MBI600	-0,04426931	0,3369127	54,29	-0,1310	0,8959
**TSWV Inoculated leaves (5 dpi)**	*SlMPK3*	Water-Mock	-0,271398484	0,1642323	56,00	-1,6530	0,104
		Water-BTH	0,640438245	0,1642323	56,00	3,9000	0,0003
		Water-MBI600	0,424682041	0,1642323	56,00	2,5860	0,0123
	SlNPR1	Water-Mock	0,006802048	0,3123818	56,00	0,0220	0,9827
		Water-BTH	1,335795172	0,3123818	56,00	4,2760	0,0001
		Water-MBI600	1,727065382	0,3123818	56,00	5,5290	<0,0001
	SlPR1b.1	Water-Mock	1,098748018	0,4294928	56,00	2,5580	0,0133
		Water-BTH	1,250713479	0,4294928	56,00	2,9120	0,0051
		Water-MBI600	0,897074054	0,4294928	56,00	2,0890	0,0413
	SlRdR1	Water-Mock	-0,510658182	0,1682427	56,00	-3,0350	0,0036
		Water-BTH	-0,916405496	0,1682427	56,00	-5,4470	<0,0001
		Water-MBI600	-0,693690869	0,1682427	56,00	-4,1230	0,0001
	SlLoxD	Water-Mock	0,792396617	0,2296402	56,00	3,4510	0,0011
		Water-BTH	-0,56690082	0,2296402	56,00	-2,4690	0,0166
		Water-MBI600	-0,154597288	0,2296402	56,00	-0,6730	0,5036
	SlCOI1	Water-Mock	-0,646259259	0,2645927	54,02	-2,4420	0,0179
		Water-BTH	-0,528908887	0,2645927	54,02	-1,9990	0,0507
		Water-MBI600	-0,478247366	0,2690932	54,03	-1,7770	0,0812

	Gene	Contrast	estimate	SE	df	t.ratio	p.value
**PVY Inoculated leaves (1 dpi)**	*SlMPK3*	Water-Mock	-0,803837705	0,512338	146,04	-1,5690	0,1188
		Water-BTH	0,141402604	0,5165428	141,54	0,2740	0,7847
		Water-MBI600	-0,001231648	0,5169027	142,15	-0,0020	0,9981
	SlNPR1	Water-Mock	-0,4473004	0,1971775	41,00	-2,2690	0,0286
		Water-BTH	-0,1899396	0,1825509	41,00	-1,0400	0,3042
		Water-MBI600	-0,4482424	0,1825509	41,00	-2,4550	0,0184
	SlPR1b.1	Water-Mock	0,5714393	0,4756406	41,00	1,2010	0,2365
		Water-BTH	-1,394033	0,4403577	41,00	-3,1660	0,0029
		Water-MBI600	-2,5972137	0,4403577	41,00	-5,8980	<0,0001
	SlRdR1	Water-Mock	-0,15620444	0,512338	146,04	-0,3050	0,7609
		Water-BTH	0,437695572	0,5323176	140,38	0,8220	0,4123
		Water-MBI600	0,097937662	0,5205632	139,47	0,1880	0,851
	SlLoxD	Water-Mock	-1,7414965	0,4042303	41,00	-4,3080	0,0001
		Water-BTH	-0,96587	0,3742445	41,00	-2,5810	0,0135
		Water-MBI600	-0,9382342	0,3742445	41,00	-2,5070	0,0162
	SlCOI1	Water-Mock	-0,03380278	0,5123380 1	46,04	-0,0660	0,9475
		Water-BTH	0,581315099	0,5170437	140,96	1,1240	0,2628
		Water-MBI600	0,688656916	0,5201741	140,06	1,3240	0,1877
**TSWV Systemic leaves (1 dpi)**	*SlMPK3*	Water-BTH	0,29348732	0,2576343	40,94	1,1390	0,2613
		Water-MBI600	0,06177086	0,2532068	40,91	0,2440	0,8085
	SlNPR1	Water-BTH	0,61869686	0,2360076	42,00	2,6220	0,0121
		Water-MBI600	1,03213475	0,2360076	42,00	4,3730	0,0001
	SlPR1b.1	Water-BTH	1,54742407	0,494422	42,00	3,1300	0,0032
		Water-MBI600	1,63030145	0,494422	42,00	3,2970	0,002
	SlRdR1	Water-BTH	0,407911	0,2585308	40,90	1,5780	0,1223
		Water-MBI600	-0,13306576	0,2541464	40,83	-0,5240	0,6034
	SlLoxD	Water-BTH	0,66765804	0,281376	42,00	2,3730	0,0223
		Water-MBI600	1,29296133	0,281376	42,00	4,5950	<0,0001
	SlCOI1	Water-BTH	-0,02094892	0,2504162	42,00	-0,0840	0,9337
		Water-MBI600	-0,18661906	0,2504162	42,00	-0,7450	0,4603
**TSWV Systemic leaves (5 dpi)**	*SlMPK3*	Water-Mock	-0,22340654	0,3077331	53,00	-0,7260	0,471
		Water-BTH	0,2875409	0,3189367	53,02	0,9020	0,3714
		Water-MBI600	0,05592442	0,3077331	53,00	0,1820	0,8565
	SlNPR1	Water-Mock	-0,95628441	0,4047325	56,00	-2,3630	0,0216
		Water-BTH	-1,45834339	0,4047325	56,00	-3,6030	0,0007
		Water-MBI600	-0,83378784	0,4047325	56,00	-2,0600	0,044
	SlPR1b.1	Water-Mock	-0,90884636	0,8529866	54,03	-1,0650	0,2914
		Water-BTH	0,85231233	0,838877	53,98	1,0160	0,3142
		Water-MBI600	-1,07570103	0,838877	53,98	-1,2820	0,2052
	SlRdR1	Water-Mock	-0,04682892	0,4441997	56,00	-0,1050	0,9164
		Water-BTH	-0,26434172	0,4441997	56,00	-0,5950	0,5542
		Water-MBI600	-0,17491746	0,4441997	56,00	-0,3940	0,6952
	SlLoxD	Water-Mock	-0,09330084	0,5503242	56,00	-0,1700	0,866
		Water-BTH	-0,95965319	0,5503242	56,00	-1,7440	0,0867
		Water-MBI600	0,23959251	0,5503242	56,00	0,4350	0,665
	SlCOI1	Water-Mock	-0,64738897	0,2563993	56,00	-2,5250	0,0144
		Water-BTH	-0,53003859	0,2563993	56,00	-2,0670	0,0433
		Water-MBI600	-0,44732397	0,2563993	56,00	-1,7450	0,0865

	Gene	Contrast	estimate	SE	df	t.ratio	p.value
**PVY Systemic leaves (1 dpi)**	*SlMPK3*	Water-Mock	-0,72276313	0,4263458	41,00	-1,6950	0,0976
		Water-BTH	-0,30273171	0,3947195	41,00	-0,7670	0,4475
		Water-MBI600	-0,15616676	0,3947195	41,00	-0,3960	0,6944
	SlNPR1	Water-Mock	0,36614845	0,2864694	41,00	1,2780	0,2084
		Water-BTH	1,0171684	0,2652191	41,00	3,8350	0,0004
		Water-MBI600	1,28016466	0,2652191	41,00	4,8270	<0,0001
	SlPR1b.1	Water-Mock	0,3840338	0,374423	41,00	1,0260	0,3111
		Water-BTH	3,88248525	0,3466484	41,00	11,2000	<0,0001
		Water-MBI600	2,29475669	0,3466484	41,00	6,6200	<0,0001
	SlRdR1	Water-Mock	-1,19357411	0,3755618	52,00	-3,1780	0,0025
		Water-BTH	-0,07571355	0,3477027	52,00	-0,2180	0,8285
		Water-MBI600	-0,60130952	0,3477027	52,00	-1,7290	0,0897
	SlLoxD	Water-Mock	-2,96829647	0,3906456	41,00	-7,5980	<0,0001
		Water-BTH	-2,3500357	0,3616676	41,00	-6,4980	<0,0001
		Water-MBI600	-0,6899926	0,3616676	41,00	-1,9080	0,0634
	SlCOI1	Water-Mock	-0,91256835	0,2826047	41,00	-3,2290	0,0024
		Water-BTH	-0,51581624	0,2616411	41,00	-1,9710	0,0554
		Water-MBI600	-0,87597194	0,2616411	41,00	-3,3480	0,0018
**PVY Systemic leaves (2 dpi)**	*SlMPK3*	Water-Mock	-0,69658316	0,3251366	41,00	-2,1420	0,0381
		Water-BTH	-0,29321862	0,301018	41,00	-0,9740	0,3357
		Water-MBI600	-0,62515648	0,301018	41,00	-2,0770	0,0441
	SlNPR1	Water-Mock	-0,8027103	0,2300992	41,00	-3,4890	0,0012
		Water-BTH	-0,68539468	0,2130304	41,00	-3,2170	0,0025
		Water-MBI600	-0,83013874	0,2130304	41,00	-3,8970	0,0004
	SlPR1b.1	Water-Mock	-0,84276439	0,3335738	41,00	-2,5260	0,0155
		Water-BTH	3,5875931	0,3088293	41,00	11,6170	<0,0001
		Water-MBI600	3,8734921	0,3088293	41,00	12,5430	<0,0001
	SlRdR1	Water-Mock	-0,8523864	0,2988289	41,00	-2,8520	0,0068
		Water-BTH	0,02486709	0,2766618	41,00	0,0900	0,9288
		Water-MBI600	-0,57948145	0,2766618	41,00	-2,0950	0,0424
	SlLoxD	Water-Mock	0,81546994	0,4456933	41,00	1,8300	0,0746
		Water-BTH	1,4904193	0,4126318	41,00	3,6120	0,0008
		Water-MBI600	1,40697589	0,4126318	41,00	3,4100	0,0015
	SlCOI1	Water-Mock	-0,54387643	0,1746661	41,00	-3,1140	0,0034
		Water-BTH	-0,34015751	0,1617094	41,00	-2,1040	0,0416
		Water-MBI600	-0,30561963	0,1617094	41,00	-1,8900	0,0659
**PVY Systemic [illegible]**	*SlMPK3*	Water-Mock	0,2450469	0,1992589	41,00	1,2300	0,2258
		Water-BTH	0,3443823	0,1844779	41,00	1,8670	0,0691
		Water-MBI600	0,5218296	0,1844779	41,00	2,8290	0,0072
	SlNPR1	Water-Mock	-0,1476448	0,3351243	41,00	-0,4410	0,6618
		Water-BTH	0,3352939	0,3102648	41,00	1,0810	0,2862
		Water-MBI600	0,5532946	0,3102648	41,00	1,7830	0,0819
	SlPR1b.1	Water-Mock	-2,3763555	0,5012737	41,00	-4,7410	<0,0001
		Water-BTH	4,1298792	0,4640893	41,00	8,8990	<0,0001

		Water-MBI600	2,8916879	0,4640893	41,00	6,2310	<0,0001
	SlRdR1	Water-Mock	-0,2072742	0,2930384	41,00	-0,7070	0,4834
		Water-BTH	1,1263691	0,2713008	41,00	4,1520	0,0002
		Water-MBI600	1,1371799	0,2713008	41,00	4,1920	0,0001

	**Gene**	**Contrast**	**estimate**	**SE**	**df**	**t.ratio**	**p.value**
	SlLoxD	Water-Mock	1,9451262	0,2133622	40,05	9,1170	<0,0001
		Water-BTH	0,8013045	0,1908828	40,01	4,1980	0,0001
		Water-MBI600	0,7114531	0,1908828	40,01	3,7270	0,0006
	SlCOI1	Water-Mock	1,3188756	0,3909583	40,43	3,3730	0,0016
		Water-BTH	1,1303468	0,3504528	40,11	3,2250	0,0025
		Water-MBI600	1,4054255	0,3504528	40,11	4,0100	0,0003

Supplementary Table S1. Contrasts and significance levels of treatments in gene expression analysis of the selected defense-related genes at different time points.

Treatment	Sample	Average Ct *SlUBI3* (±Stdev)	Average Ct P3 (±Stdev)
Water	1	19,6 (±0,2)	25,3 (±0,2)
	2	19,9 (±0,3)	29,8 (±0,2)
	3	19,4 (±0,3)	27,4 (±0,4)
	4	19,3 (±0,1)	28,5 (±0,2)
BTH	1	18,2 (±0,2)	35,6 (±0,2)
	2	18,9 (±0,3)	33,8 (±0,1)
	3	19,3 (±0,2)	34,2 (±0,6)
	4	18,9 (±0,3)	30,6 (±0,2)
MBI600	1	20,0 (±0,2)	36,8 (±0,2)
	2	20,4 (±0,2)	Undetectable
	3	21,4 (±0,2)	33,1 (±0,4)
	4	19,8 (±0,02)	37,2 (±0,4)
Healthy Tomato		20,1 (±0,4)	Undetectable

Supplementary Table S2. RT-qPCR analysis of the apical non-inoculated leaves of plants inoculated with *Potato virus Y* (PVY), at five days post inoculation. The quantification of the virus was performed according to the Ct values for the P3 genomic region of PVY.

Primer	Sequence (5´-3´)	Locus
MPK3-F[4]	CATGATGCAGCTGATGAACCA	Solyc06g005170.2
MPK3-R[4]	GCATATTCAGGATTCAACGCC	
RdR1-F[4]	GCCGACAACATAAGTGCTGACA	Solyc05g007510.2
RdR1-R[4]	CCCGCAGTTGGACAATATCA	
COI1-F[56]	TTCTGCATATTTCTCGTTCCTGCAA	Solyc05g052620.2
COI1-R[56]	GCTAGTTCATGTGCCCATTCTCCAT	
LoxD-F[57]	CCATCCTCACCACCCTCATC	Solyc03g122340.2
LoxD-R[57]	TACTCGGGATCGTTCTCGTC	
PR1b-F[21]	GGTCGGGCACGTTGCA	Solyc00g174340.1
PR1b-R[21]	GATCCAGTTGCCTACAGGACATA	
NPR1-F	TGTGGGAAAGATAGCAGCACG	Solyc07g040690.2
NPR1-R	GTCCACACAAACACACACATC	
UBI3-F[52]	GCCGACTACAACATCCAGAAGG	Solyc01g056940.2
UBI3-R[52]	TGCAACACAGCGAGCTTAACC	
PVYSON41-F[4]	CACGAACACCAGTGAGGGCT	P3 locus
PVYSON41-R[4]	CGGTGGTGTGCCTCTCTGT	

Supplementary Table S3. Description of forward (F) and reverse (R) primers used in RT-qPCR for *Potato virus Y* (PVY) quantification and the expression analysis of genes considered as markers of direct defense responses and the priming mechanism.
